# Fast Dynamic Vehicle Detection in Road Scenarios Based on Pose Estimation with Convex-Hull Model

**DOI:** 10.3390/s19143136

**Published:** 2019-07-17

**Authors:** Kaiqi Liu, Jianqiang Wang

**Affiliations:** School of Vehicle and Mobility, Tsinghua University, Beijing 100084, China

**Keywords:** autonomous vehicle, environmental perception, Lidar, dynamic vehicle detection

## Abstract

Dynamic vehicle detection is of great significance for the safety of autonomous vehicles and the formulation of subsequent driving strategies. A pose-estimation algorithm, namely, the pose estimation with convex-hull model (PE-CHM), is proposed in this paper, and introduced in the dynamic vehicle detection system. In PE-CHM, the convex hull of the object’s point-clouds is first extracted and the most fitted bounding box is determined by a multifactor objective function. Next, the center position of the target is inferred according to the location and direction of the target. With the obtained bounding box and the position inference, the pose of the target is determined, which reduces the interference of the missing contour on pose estimation. Finally, three experiments were performed to validate the performance of the proposed PE-CHM method. Compared with several typical model-based methods, PE-CHM can implement dynamic vehicle detection faster, which reduces the amount of calculation on the basis of ensuring detection efficiency.

## 1. Introduction

There are generally static and moving obstacles in traffic environments. For autonomous vehicles in road scenario, since moving objects have their own movement state and motion direction, their motion randomness affects the behavior decision of an autonomous vehicle, which poses a greater threat to traffic safety. Therefore, the detection of moving vehicles is an important task [1,2,3], and is considered in this work.

For vehicle detection, camera and Lidar are commonly used sensors. The camera has the closet structure to that of the human eye, and can reflect features such as the color, texture, and edges of the target [4,5,6]. A dynamic object detection method in monitoring scenario with fixed cameras has been proposed [7]. The method models each pixel as mixture of Gaussians and can deal with slow lighting changes and multimodal distributions caused by shadows, specularities, swaying branches and other troublesome features of the real world. But this kind of method is not suitable to detect the moving objects for the mobile platform. In [8], a front-vehicle detection with oriented fast and rotated brief (ORB) matching method and spatial constraints was proposed, which had a good performance in both vehicle detection efficiency and effectiveness. However, it is difficult to obtain the accurate distance and contour size of the target with a monocular camera. The distance information is of great significance in the formulation of driving strategies for autonomous vehicles, which can easily be obtained with a Lidar sensor.

There are many useful methods to detect vehicles with Lidar [9]. Among these detection methods, the training-based and model-based methods are two common vehicle detection methods [10,11].

### 1.1. Training-Based Method

The training-based method usually extracts features from the objects with point-clouds data and employs classifiers to detect both static and dynamic vehicles. In [12], an adaptive mean shift was employed to segment the point-clouds. From the segments, five object attributes, area, elongatedness, planarity, vertical position, and range, were utilized to classify objects into vehicles and nonvehicles with a support vector machine (SVM). In [13], a new approach for object classification was proposed. A convolutional neural network was employed to preliminarily classify the objects, which were refined with contextual features considering possible expected scene topologies. A dataset with 1485 objects was applied to test the method, and the F-score average reached 89%. In [14], three descriptors combined with an SVM were utilized for vehicle classification. The three features were the length and width of the approximated 2D bounding box derived from the convex hull, the four radius values of the 3D spheres, the radius difference between the frontal and the back sphere pairs, and the difference between the concave side profile hull of the candidate vehicle and the prototype shape. The average F-score reached 86%. In [15], multiple sensors were utilized to detect and track moving objects. Lidar are applied to detect the dynamic objects with cells change and determine the regions of interest (ROI). The Histogram of Oriented Gradient (HOG) descriptor from visual images as well as discrete Adaboost is employed to classify the objects. With the fused information, the detection in highway scenario reached 97.8%. In [16], a real-time 3D vehicle detection method was proposed. A pre-RoI-pooling convolution technique and pose-sensitive feature map were utilized to improve computation efficiency and detection accuracy. The method could complete detection within 0.09 s. In [17], three features, including the 2D distance of the cluster, total number of points and the direction of the clustered points’ distribution, are utilized to classify the vehicle and pedestrian combined with the backpropagation artificial neural network (BP-ANN). The detection and tracking of the proposed roadside Lidar data processing procedure are above 95%.

In general, the training-based method has the ability to achieve good performance in vehicle detection. However, the method requires large amounts of data for model training, while complex and variable scenarios could hardly afford the adequate training data. At the same time, the training process usually costs a lot of computing resources. These constitute the main limitations of the training-based method.

### 1.2. Model-Based Method

In [18], an object-based hierarchical two-level model is proposed for the joint probabilistic extraction of vehicles with aerial Lidar point-clouds that are relatively dense compared with common vehicle Lidar, such as Velodyne HDL-64E. A dataset with 1009 vehicles was applied in the experiment, 97% (F-score) of which could be detected with the proposed method. In [19], a new detection method of line segments in scanning laser-range measurements was proposed that could be especially applied to vehicle detection. Feature extraction could be summed up in the detection of triangles in the cumulative function and property estimation. A model-based joint detection and tracking method was proposed in [20], which is a track-before-detect method. The method improved estimation-accuracy performance, robustness to false alarms, and track continuity based on a bounding-box model. In [21], the L-shape model was proposed for vehicle detection and tracking, which can overcome the appearance change problem caused by Lidar. However, precise corner point position and heading angle extraction are required for the best performance. The reference [11] also used the minimum rectangle area with L-shape cloud fitting for box-fitting, which detect and track the objects in a close area.

In [22], a likelihood-field-based vehicle measurement model as well as the pose estimation with modified scaling series (PE-MSS) algorithm were proposed to detect the dynamic vehicles. Motivated by the work in [22], we used the information obtained from the point set registration with the process of pose estimation and the proposed pose estimation based on coherent point drift (PE-CPD) algorithm [10],which did not bring an additional computation burden to the algorithm and improved detection efficiency. Although the detection method was proven to have the ability to complete the detection of a scenario that contained a single vehicle within 100 ms under the MATLAB platform, the model-fitting process with likelihood-field-based model still required a large amount of computation and consumed many computing resources.

The main objective of this work was to propose a dynamic vehicle detection system with Lidar point-clouds that could quickly and accurately detect dynamic vehicles in the scenarios, thereby improving the ability for autonomous vehicles to sense environmental hazards. In this work, our contributions mainly focus on the proposed vehicle-pose estimation algorithm based on a point-cloud convex-hull model and position inference, and  estimation results were introduced into the process of dynamic vehicle detection. Compared with the existing methods, the proposed pose-estimation method requires low computation and is robust to the environment, which reduces the amount of computation while maintaining good detection efficiency.

The remainder of the paper is organized as follows. In Section 2, the process of pose estimation with convex-hull model (PE-CHM) is described in detail. Based on PE-CHM, the dynamic vehicle detection system is introduced in Section 3. Experiments with several comparative methods were implemented and are analyzed in Section 4. Finally, concluding remarks are shown in Section 5.

## 2. Pose Estimation with CHM

Pose estimation of the target can be utilized for target detection [10,22]. The target pose in this work is denoted by $\left(C_{x},C_{y},\theta \right)$, in which $\left(C_{x},C_{y}\right)$ is the center position of the target in bird-view projection, and θ is the yaw angle. The process of the proposed PE-CHM method mainly includes two processes, convex-hull model fitting and position inference, which are detailed below.

### 2.1. Convex-Hull Model Fitting

**Figure 1:**
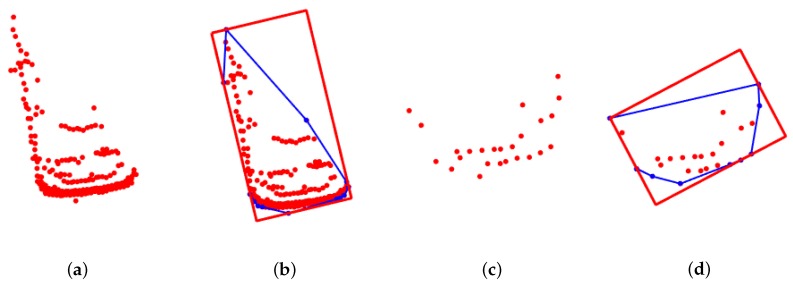
Convex-hull model-fitting results with multifactor objective function. (**a**,**c**) Original point-clouds of two vehicles. (**b**,**d**) Corresponding fitted bounding box with the convex-hull model. Blue lines show the convex hull of the point-cloud set.

Since most of the Lidar point-clouds come from the object contour, a point-cloud convex-hull model with a multifactor objective function is proposed to fit the point-cloud set. The model-fitting results of two vehicles are shown in Figure 1. The 3D point-clouds are projected on the *x*-*y* plane regardless of the height dimension. The detailed modeling process is shown in Algorithm 1.

**Figure 2:**
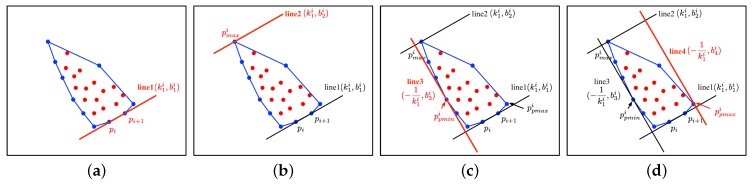
The determination process of the bounding box. (**a**–**d**) indicate the determination process of line 1 to line 4, respectively. The dots denote the point-clouds. The polygon represented by the blue lines denote the convex hull of this set of point-clouds.

The input of Algorithm 1 includes the point-clouds of object *P* and its approximate direction vector $D_{y}$ that is obtained by the positions of the associated objects. First, the convex hull of the point-clouds set is determined [23]. The selected *K* points surround all the point-clouds in set *P*. As shown in Figure 2a, the two adjacent points $p_{i}=\left(x_{i},y_{i}\right)$ and $p_{i+1}=\left(x_{i+1},y_{i+1}\right),i=1,...,m-1$ in set *K* are sequentially selected to determine the equation of line 1 with slope $k_{1}^{i}$ and intercept $b_{1}^{i}$, in which *m* is the number of points in *K*. The calculation process is shown in Equation (1).

(1) $$k_{1}^{i}=\frac{y_{i+1}-y_{i}}{x_{i+1}-x_{i}},\;\;\;\;\;\;\;\;\;\;b_{1}^{i}=\frac{x_{i+1}y_{i}-y_{i+1}x_{i}}{x_{i+1}-x_{i}}$$

**Algorithm 1** Convex-hull model (CHM) fitting.**Input:**$P={\left(p_{1},p_{2},...,p_{n}\right)}^{T}$, $D_{y}$; 1:$K=\mathrm{ConvexHull}(P_{x},P_{y})$;2:**for**i=1,...,m−1**do**3:  $\left(k_{1}^{i},b_{1}^{i}\right)=\mathrm{ParameterofLine}\left(p_{i},p_{i+1}\right)$;4:  $D^{i}=\mathrm{DistancetoLine}\left(K,k_{1}^{i},b_{1}^{i}\right)$;5:  $p_{max}^{i}=\left\{p_{j}|d_{j}=max\left(D^{i}\right)\right\}$;6:  $b_{2}^{i}=\mathrm{ParameterofLine}\left(p_{max}^{i},k_{1}^{i}\right)$;7:  $X_{p}^{i}=\mathrm{ProjectiontoLine}\left(k_{1}^{i},b_{1}^{i}\right)$;8:  $p_{pmax}^{i}=\left\{p_{j}|x_{pj}=max\left(X_{p}^{i}\right)\right\}$; $p_{pmin}^{i}=\left\{p_{j}|x_{pj}=min\left(X_{p}^{i}\right)\right\}$;9:  $b_{3}^{i}=\mathrm{ParameterofLine}\left(p_{pmin}^{i},-\frac{1}{k_{1}^{i}}\right)$; $b_{4}^{i}=\mathrm{ParameterofLine}\left(p_{pmax}^{i},-\frac{1}{k_{1}^{i}}\right)$;10:  $R\left(i\right)=\left[k_{1}^{i},b_{1}^{i},b_{2}^{i},b_{3}^{i},b_{4}^{i}\right]$;11:  A(i)=AreaofBoundingBox(R(i));12:  D(i)=SumofMinDistancetoEdges(K,R(i));13:  M(i)=MaxofMinDistancetoEdges(K,R(i));14:  $\Theta \left(i\right)=\mathrm{SelectYawAngleDif}\left(D_{y},k_{1}^{i}\right)$;15:**end for**16:$\left(R_{optimal},\theta \right)=\mathrm{SelectOptimalParameter}\left(R,A,D,M,\Theta \right)$;17:$\left(x_{rect},y_{rect}\right)=\mathrm{IntersectionofLine}\left(R_{optimal}\right)$;**Output:**$\left(x_{rect},y_{rect},\theta \right)$;

Next, point $p_{max}^{i}=\left(x_{max}^{i},y_{max}^{i}\right)$ in *K* with the farthest distance from line 1 is selected. According to slope $k_{1}$ and the coordinates of $p_{max}$, intercept $b_{2}^{i}$ of line 2 is calculated, which is shown in Figure 2b.

(2) $$b_{2}^{i}=y_{\max}^{i}-x_{\max}^{i}k_{1}^{i}.$$

Subsequently, all points in set *K* are projected to line 1, and the *x* value of each projected point is obtained, which is represented as $X_{p}^{i}$.

(3) $$X_{p}^{i}=\frac{K_{x}+k_{1}^{i}K_{y}-b_{1}^{i}k_{1}^{i}}{{\left(k_{1}^{i}\right)}^{2}+1},$$

where $K_{x}$ and $K_{y}$ represent the *x* values and *y* values of the points in *K*. The points with the minimum and maximum projected *x* values $p_{pmax}^{i}=\left(x_{pmax}^{i},y_{pmax}^{i}\right),x_{pmax}^{i}=\max\left(X_{p}^{i}\right)$ and $p_{pmin}^{i}=\left(x_{pmin}^{i},y_{pmin}^{i}\right),x_{pmin}^{i}=\min\left(X_{p}^{i}\right)$ are selected to confirm the other two edges of the box with slope $-\frac{1}{k_{1}^{i}}$, which are shown in Figure 2c,d, respectively. The parameters are calculated with Equation (4).

(4) $$b_{3}^{i}=y_{pmin}^{i}+\frac{1}{k_{1}^{i}}x_{pmin}^{i},\;\;\;\;\;\;\;\;b_{4}^{i}=y_{pmax}^{i}+\frac{1}{k_{1}^{i}}x_{pmax}^{i}.$$

All parameters of the four edges, including $k_{1}^{i}$ and $b_{1}^{i}$-$b_{4}^{i}$, are recorded in R. The area of the box determined by the four edges is recorded in A. The sum value and maximum value of minimum distance of each point in *K* from the edges of the box were calculated and recorded in D and M, respectively.

When the object in time step *k* was associated with the object at time step k−1, the approximate direction vector $D_{y}$ can be obtained with the position of the object at these two time steps. The geometric center of the object at time step k−1 and *k* is denoted by $\left(c_{x}^{k-1},c_{y}^{k-1}\right)$ and $\left(c_{x}^{k},c_{y}^{k}\right)$, respectively. $D_{y}$ is calculated with Equation (5).

(5) $$D_{y}=\left(c_{x}^{k}-c_{x}^{k-1},c_{y}^{k}-c_{y}^{k-1}\right).$$

If the object is not associated with other objects in adjacent scan frames, the vector is set to be empty. Vector $D_{y}$ and slopes of bounding-box edges $k_{1}^{i}$ and $-\frac{1}{k_{1}^{i}}$ are utilized to determine the object’s approximate pose. The angles between vector $D_{y}$ and the two straight lines whose slopes are $k_{1}^{i}$ and $-\frac{1}{k_{1}^{i}}$ are calculated, respectively, and the smaller angle in $\Delta \theta_{1}$ and $\Delta \theta_{2}$ is stored in Θ. The angle difference is calculated with Equation (6).

(6) $$\Delta \theta_{1}=\mathrm{atan}\left(\left|\frac{k_{1}^{i}-k_{D_{y}}}{1+k_{1}^{i}\cdot k_{D_{y}}}\right|\right),\Delta \theta_{2}=\mathrm{atan}\left(\left|\frac{1+k_{1}^{i}\cdot k_{D_{y}}}{k_{1}^{i}-k_{D_{y}}}\right|\right),$$

where $k_{D_{y}}$ is the slope of vector $D_{y}$.

Then, points $p_{i+1}$ and $p_{i+2}$ are utilized in the next for-loop. After all the points in set *K* have been applied to construct the bounding box, an objective function is proposed to select the optimal parameters. Objective function *F* is show below.

(7) $$F=\sum_{i=1}^{4}\omega_{i}\cdot \frac{f_{i}-\min\left(f_{i}\right)}{\max\left(f_{i}\right)-\min\left(f_{i}\right)},f_{i}\in \left\{A,D,M,\Theta \right\},$$

where $\omega_{i}$ denotes the weights of each factor, $\sum_{i=1}^{4}\omega_{i}=1$. $f_{i}$ denotes one of the four impact factors. Set D and set M represent the sum and maximum value of the minimum distance of the points in set *K* from the bounding box, respectively. Set A stores the area value of each bounding box. Set Θ stores the angle between the direction vector and the yaw angle of each bounding box. In order to comprehensively consider the influence of the factors of different measurement spaces, the maximum and minimum values of the set are utilized to normalize the measurements. The parameters of the bounding box with the smallest *F* value are selected as the optimal parameters, and the corresponding bounding box is defined as the fittest box.

### 2.2. Position Inference

The convex-hull model in the last section can obtain the yaw angle of the target. To obtain complete pose information, the position of the target needs to be determined. From [22], the center of the bounding box with a minimum area is utilized as the initial center position. Then, the center position is iteratively calculated by model fitting. The reason that the center of the bounding box is not taken as the position of the target is that the obtained center position is inaccurate, as the contour of target is missing. As shown in Figure 1c, the vehicle is far from the Lidar and only one short side can be seen. It is obvious that the center of the bounding box in Figure 1d is not the actual center position of the vehicle, and there is a large deviation between them. In this section, the position-inference process is applied to obtain its center position without iteration.

**Figure 3:**
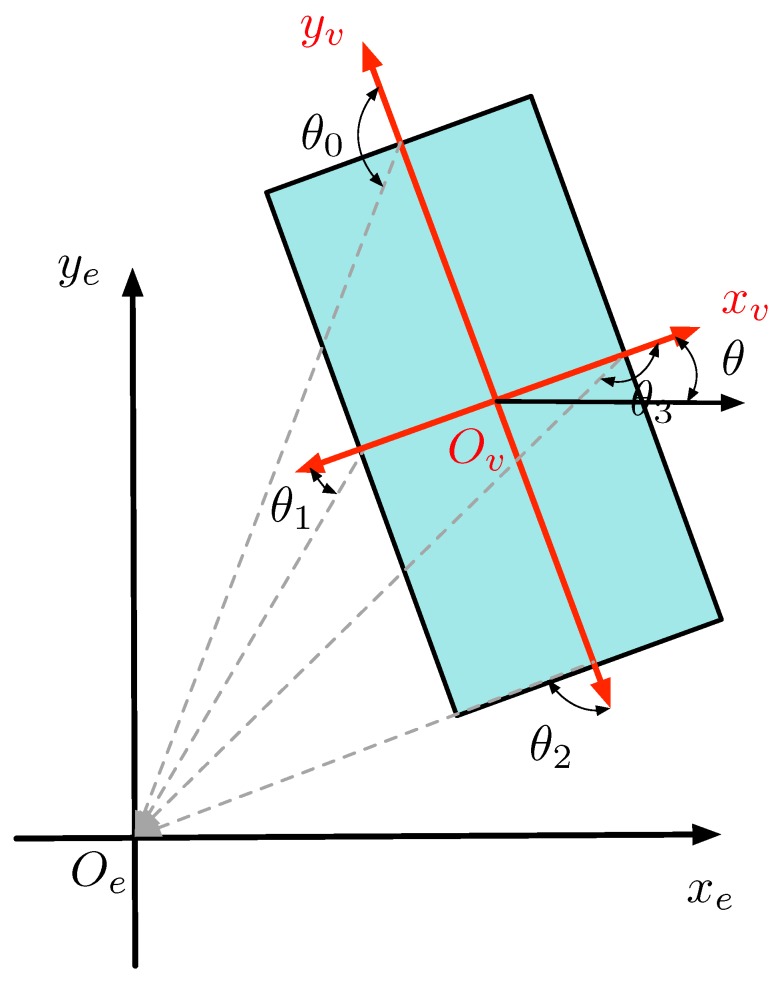
Rectangular model of the vehicle.

Position inference uses a fixed model size to determine the center of the target based on its position relative to Lidar. Figure 3 shows the rectangular model of the vehicle. $O_{e}x_{e}y_{e}$ is the Lidar coordinate system. $y_{e}$ axis points forward and $x_{e}$ points to the right side of Lidar. $O_{v}x_{v}y_{v}$ is the coordinate system of the target vehicle with $y_{v}$ points to the moving direction of target vehicle and $x_{v}$ points to its right side. Angle θ between $x_{v}$ and $x_{e}$ is defined as the yaw angle of the target vehicle.

The obtained convex-hull model was employed to determine the visible edges of the targets. As shown in Figure 3, the vectors that point from the center of model to the center of each side constitute a normal vector set, and the vectors that point from the center of each side to the origin of the Lidar constitute the orientation vector set. For each edge, the angle between the orientation vector and normal vector show whether the edge can be seen under the perspective of the Lidar [22]. If the angle is greater than $\frac{\pi }{2}$, the edge is invisible; otherwise, the edge is visible.

Before inference, the geometrical center of the point-clouds is momentarily employed. The normal vector and orientation vector of each edge in the bounding box are obtained, by which the visible edges are determined. Generally, there are two edges visible in the scenario except when the object is directly in front of the Lidar, so that the angle between the normal vector and the orientation vector is 0, which is a small probability event and almost nonexistent in practice. It is assumed that there are theoretically always two visible edges, and the intersection point is denoted by $p_{itsc}$.

**Figure 4:**
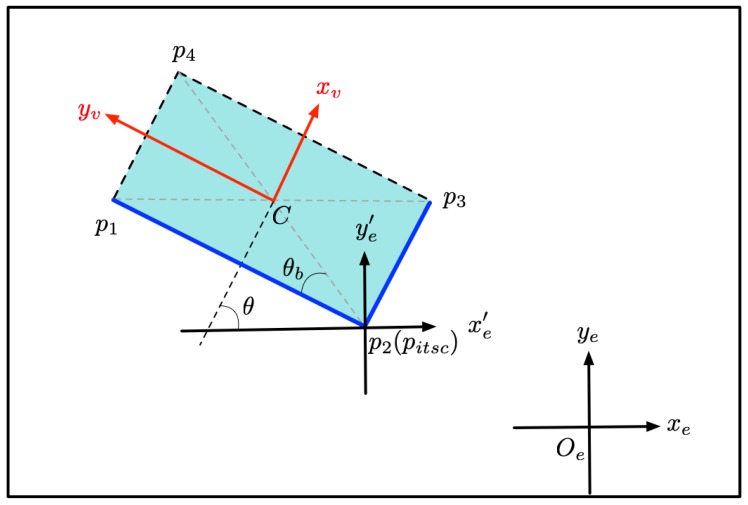
Center-coordinate determination with position inference. Two blue lines, visible edges. $x_{v}$ and $y_{v}$, two axes of object’s local coordinate system. $x_{e}$ and $y_{e}$, two axes of global Lidar coordinate system. $x_{e}^{\prime }$ and $y_{e}^{\prime }$, axes with $p_{itsc}$ as the origin and parallel to $x_{e}$ and $y_{e}$, respectively.

Figure 4 shows an example of position inference. Lines $p_{1}p_{2}$ and $p_{2}p_{3}$ are the two visible edges. The angle θ is its yaw angle. The angles between $y_{v}$ and lines $p_{1}p_{2}$ and $p_{2}p_{3}$ are calculated, respectively. As can be seen from the Figure 4, the edge with the larger angle is the short edge, and the edge with the smaller angle is the long edge. The size of the model is assumed to be constant. The length and width of the model are set to *L* and *W*. Angle $\theta_{b}$ is determined depending on the relationship between the visible edge and $y_{v}$ with Equation (8).

(8) $$\theta_{b}=\left\{\begin{array}{cc} \mathrm{atan}(\frac{W}{L}), & \mathrm{if}\;\;\;\;\langle \vec{p_{2}p_{1}},y_{v}\rangle \leq \langle \vec{p_{2}p_{3}},y_{v}\rangle \\ \mathrm{atan}(\frac{L}{W}), & \mathrm{otherwise} \end{array}\right..$$

After calculating intersection $p_{itsc}$ with the line equations of the two visible edges and angle $\theta_{b}$, the coordinates of the model center can be obtained with Equations (9) and (10).

(9) $$c_{x}=x_{p_{itsc}}+\frac{\sqrt{L^{2}+W^{2}}}{2}\cdot \cos\left(\langle \vec{p_{2}p_{1}},x_{e}^{\prime }\rangle -\theta_{b}\right)$$

(10) $$c_{y}=y_{p_{itsc}}+\frac{\sqrt{L^{2}+W^{2}}}{2}\cdot \sin\left(\langle \vec{p_{2}p_{1}},x_{e}^{\prime }\rangle -\theta_{b}\right)$$

where $\left(x_{p_{itsc}},y_{p_{itsc}}\right)$ is the coordinate of intersection point $p_{itsc}$, $\langle \vec{p_{2}p_{1}},x_{e}^{\prime }\rangle$ denotes the angle between vector $\vec{p_{2}p_{1}}$ and $x_{e}^{\prime }$.

### 2.3. Pose Estimation

The pose-estimation process uses the convex-hull model and position inference to determine the pose of target. The detailed description of pose estimation is described in Algorithm 2.

The inputs of Algorithm 2 include the point-cloud coordinates of the object and approximate direct vector $D_{y}$. The output is the center position and yaw angle of the target.

First, CHM fitting in Algorithm 1 was implemented to select the optimal bounding box. The center of the box can be obtained, and the theoretical visible edges were determined with the normal vector and orientation vector of the model. Then, the intersection point of the two visible edges is calculated. With the fixed size of the vehicle rectangular model and the obtained yaw angle, the center position of the target is finally infered.

**Algorithm 2** Pose estimation with convex-hull model (PE-CHM).**Input:**$P={\left(p_{1},p_{2},...,p_{n}\right)}^{T}$, $D_{y}$; 1:$\left(x_{rect},y_{rect},\theta \right)=\mathrm{CHM}\_\mathrm{Fitting}\left(P,D_{y}\right)$;2:$c_{x}^{m}=\mathrm{mean}\left(x_{rect}\right)$, $c_{y}^{m}=\mathrm{mean}\left(y_{rect}\right)$;3:$N^{m}=\mathrm{NormalVector}\left(x_{rect},y_{rect},c_{x}^{m},c_{y}^{m}\right)$;4:$O^{m}=\mathrm{OrientationVector}\left(x_{rect},y_{rect}\right)$;5:$E_{v}=\mathrm{FindVisibleEdge}\left(N^{m},O^{m}\right)$;6:$p_{itsc}=\mathrm{FindIntersect}\left(E_{v}\right)$;7:$\left(c_{x},c_{y}\right)=\mathrm{PositionInference}\left(\theta ,D_{y},p_{itsc}\right)$;**Output:**$\left(c_{x},c_{y},\theta \right)$;

## 3. Dynamic Vehicle Detection

**Figure 5:**
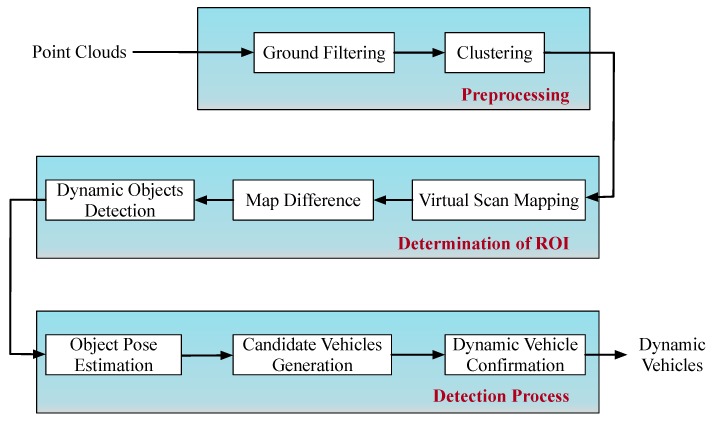
Framework of the dynamic vehicle detection based on pose estimation with convex-hull model (PE-CHM).

In order to detect dynamic vehicles fast and accurately, a detection system with PE-CHM is proposed in this section. The framework of the detection process is shown in Figure 5.

The detection system mainly included three parts. First, point-clouds are preprocessed to reduce the amount of data, and clustered based on the 3D point-cloud characteristics. Second, a virtual-scan map is constructed to determine the region of interest, and the dynamic objects are extracted from the clustering results. At this stage, the 3D coordinates of the point-clouds are projected onto the 2D *x*-*y* plane for subsequent processing. Third, the poses of the extracted objects are estimated, combined with motion consistency, and the object is judged as a vehicle or nonvehicle. The detailed process is shown below.

### 3.1. Preprocessing

The number of point-clouds received by the Lidar is usually large, and a large percentage of the point-clouds come from the ground. In order to reduce the number of point-clouds to be processed and eliminate the interference of the measured outliers, the original point-clouds need to be preprocessed before detection [24]. The preprocessing process in this paper includes ground filtering and point-cloud clustering.

Many ground-filtering methods of point-clouds have been proposed [25,26,27,28]. Generally, road surfaces are relatively flat. Considering operation time, the extended Kalman filter (EKF)-based ground-filtering algorithm in [28] is utilized. Subsequently, the radially bounded nearest neighbor (RBNN) algorithm [29] is applied to cluster point-clouds above the ground. Among clustering results, clusters with few point-clouds are eliminated.

### 3.2. ROI Determination

Dynamic objects are detected from the preprocessing results, which constitute the ROI and avoid the complexity of detecting vehicles in the entire point clouds. The ROI extraction process includes virtual-scan mapping, virtual-scan difference, and dynamic-object detection.

#### 3.2.1. Virtual-Scan Mapping

**Figure 6:**
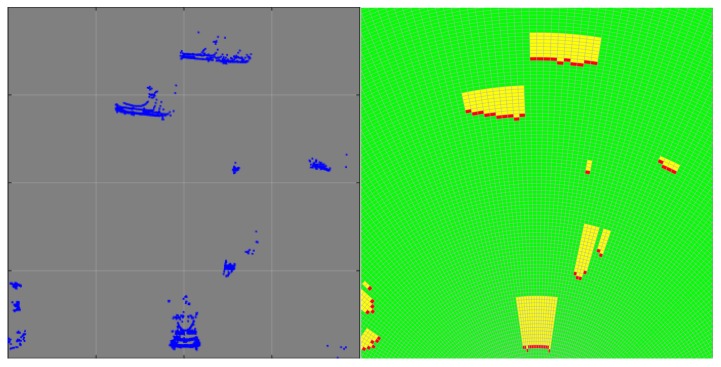
Virtual-scan mapping. (**left**) Point-clouds; (**right**), corresponding virtual scan. States of grids in green, red, and yellow are free, occupied, and occluded, respectively.

After preprocessing, the remained point-clouds above the ground are projected to the virtual-scan map for dynamic-object detection. The virtual-scan map is a polar grid representation of the point-clouds [10]. As shown in Figure 6, the polar grid map is divided into grids with the same center angles and radial lengths. The state of each grid is determined by the number and position of point-clouds. The grid without point-clouds projected is defined as free, as shown by the green grids in Figure 6. In each sector divided by the same center angle, the grid closest to the Lidar in the place where the object is located is defined as the occupied state, which are shown with the red color. The states of the grids between the occupied grid and the farthest grid where the object is located are defined as the occluded states, which are shown in yellow.

#### 3.2.2. Virtual-Scan Difference

Since the origin of the point-cloud coordinates is the Lidar location, the coordinate systems of the two consecutive point-clouds should be unified before calculating the virtual-scan difference when the Lidar is on a mobile platform. The transformation process with the information received by GPS-ISN is as follows.

(11) $$T_{c}={\left[\begin{array}{cc} R^{k} & t^{k}\\ 0 & 1 \end{array}\right]}^{-1}\cdot \left[\begin{array}{cc} R^{k-1} & t^{k-1}\\ 0 & 1 \end{array}\right]$$

(12) $$R^{k}=R\left(\gamma^{k}\right)*R\left(\varphi^{k}\right)*R\left(\phi^{k}\right),$$

where k−1 and *k* represent two consecutive comments. *R* and *t* denote the rotation matrix and translation vector. γ, φ, and ϕ denote roll angle, pitch angle, and yaw angle of the ego vehicle, respectively. With the transformation matrix $T_{c}$, the coordinates of the point-clouds in time step k−1 are transformed into the coordinate system in time step *k*. The transformed coordinates of $P_{k-1}$ are represented as $P_{k-1}^{*}$.

(13) $$P_{k-1}^{*}=T_{c}\cdot P_{k-1}.$$

After converting the point-cloud coordinates in time step k−1 to the coordinate system in time step *k*, virtual-scan mapping is performed on the two frames of the point-clouds, respectively, and the map difference is implemented.

#### 3.2.3. Dynamic-Object Detection

The difference of two consecutive virtual scans is applied to detect the dynamic objects in the scenario [15]. Dynamic objects are detected according to the change of the grid state. Adaptive threshold $T_{do}$ of the changed grids for object *o* is employed in detection [10].

(14) $$T_{do}=\left\lceil \frac{W}{A_{r}\cdot ∥o∥}\right\rceil ,$$

in which *W* denotes the width of the vehicle. $A_{r}$ represents the angular resolution of the virtual scan. $∥\cdot ∥$ is the Euclidean distance between object and Lidar. $\left\lceil \cdot \right\rceil$ is the rounding-up operation. According to the virtual-scan difference and adaptive detection threshold, dynamic objects in the scenario are detected, which become the ROI for subsequent dynamic vehicle detection.

### 3.3. Dynamic Vehicle Detection

**Figure 7:**
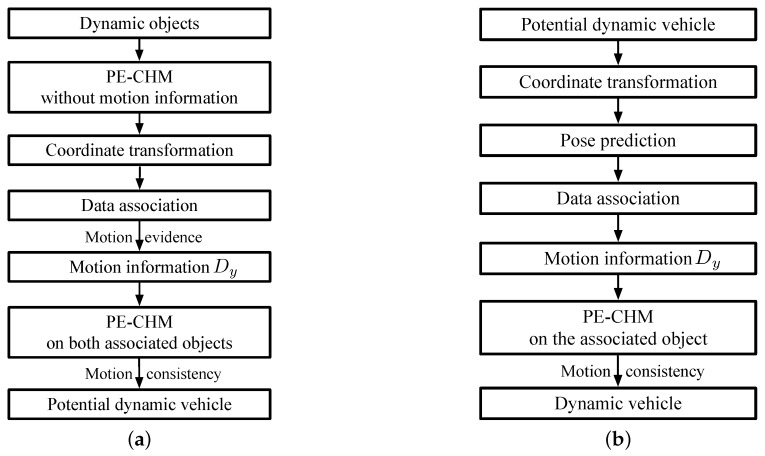
Flowchart of dynamic vehicle detection. (**a**) Potential dynamic vehicle detection. (**b**) Dynamic vehicle determination.

In this section, the dynamic vehicle detection system is detailed. The flowchart of the first stage in the detection process is shown in Figure 7a.

As shown in Figure 7a, after obtaining the dynamic objects in Section 3.2.3 at time step *k*, the pose of the object is determined by PE-CHM with $D_{y}=\emptyset$, and the obtained yaw angle θ contains two angles that differ by 90 degrees. Subsequently, the point-clouds at time step k−1 are transformed into the coordinate system at time step *k*. Along the direction of yaw angle, velocity is evenly sampled in the range of $\left[v_{1}^{s},v_{2}^{s}\right]$ to find the associated object at time step k−1. When the state of the objects at time step *k* and k−1 satisfy motion theory [22], they are associated as the same object. Motion theory determines whether objects are associated by inspecting the grid change between the objects’ positions in the virtual scan at consecutive time steps. Approximate direction vector $D_{y}$ is obtained by the associated objects. With the direct vector, the poses of the associated objects at time step k−1 and *k* are determined with PE-CHM. Motion consistency [22] is then applied to judge the dynamic objects. Motion consistency means that changes of velocity and yaw angle should be small. After the judgment of motion consistency, potential vehicles are detected.

Subsequently, as can be seen in Figure 7b, at time step k+1, the point-cloud coordinates of time step *k* are transformed into the coordinate system at time step k+1. The pose of the potential vehicles is applied to select their associated objects. The poses are predicted according to the obtained $D_{y}$, yaw angle, and velocity. Objects near the predicted poses are applied to get new approximate direct vector $D_{y}$, and are modeled with PE-CHM. If the associated objects at time step k−1 to k+1 satisfy motion consistency, the object at time step k+1 is first detected as a dynamic vehicle.

At time step k+2, for the detected dynamic vehicle, a similar process to Figure 7b is employed to update the state of the detected vehicle. If motion consistency is not satisfied, the vehicle is eliminated.

### 3.4. Computational Complexity

The purpose of this work was to develop a dynamic vehicle detection system that has the ability to detect targets quickly and accurately. In this section, the complexity of the detection process is analyzed compared with the method based on the likelihood-field-based model, which has been proven to perform well in dynamic vehicle detection [10]. Considering that the framework of the detection process in [10] and the process in this work are similar, mainly the complexity of the pose-estimation process is analyzed in this section.

By analyzing the process of Algorithm 2 and the process in [10], the largest computational complexity is concentrated on the solution of the point-cloud convex hull [23]. Therefore, in terms of complexity, their operation complexity is in the same order. Even so, the proposed method has a large advantage in reducing computing resources. Specifically, in Algorithms 1 and 2, the main operation focuses on the for loop to find the best bounding box. It is assumed that the convex-hull process obtains *m* points that can surround the objects, whose complexity is O(m), in most cases m<n, and only when the target point-clouds returned from a single layer, m=n. In PE-CPD, meanwhile, calculation mainly focuses on the measurement model-fitting process. The integral value of each point needs to be calculated. Though the erf function was utilized instead of the integral operation for reducing computational complexity, the computational complexity of this process is still O(n). At the same time, the introduction of the CPD process also brought computation complexity of O(n). Therefore, in addition to O(nlogn) that is needed by the convex hull, PE-CHM additionally brings computational complexity of O(m)+O(1), while PE-CPD brings computational complexity of 2O(n)+O(1). Therefore, PE-CHM brings a reduction in the amount of calculation.

## 4. Experiments and Results

In this paper, three experiments were performed to validate the performance of the proposed pose-estimation algorithm and dynamic vehicle detection system. The KITTI dataset [30] was employed in the experiments. In the KITTI dataset, the autonomous driving platform was equipped with two high-resolution color cameras, two grayscale video cameras, a Velodyne HDL-64E, and a GPS localization system. The provided label set of the Velodyne point-clouds was located within the view of the optical sensors, that is, in front of the ego vehicle. Therefore, only the point-clouds in front of the ego vehicle were processed in this paper. Considering that the provided labels are incomplete, the labels of each frame of the point-clouds were manually corrected. The angular resolution in virtual scan map is set 1 degree. The velocity range of $\left[v_{1}^{s},v_{2}^{s}\right]$ when associating objects is set to [−35,35] (m/s). The sizes of vehicle model are set to L=4.8 m and W=1.8 m.

### 4.1. Pose-Estimation Accuracy

**Figure 8:**
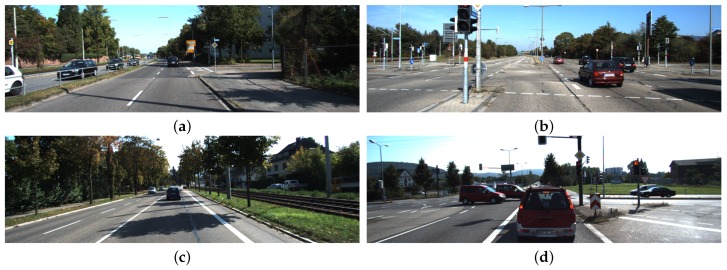
The optical images of KITTI datasets. (**a**–**d**) represent the scenarios in 0014, 0018, 0056 and 0057 datasets, respectively.

**Figure 9:**
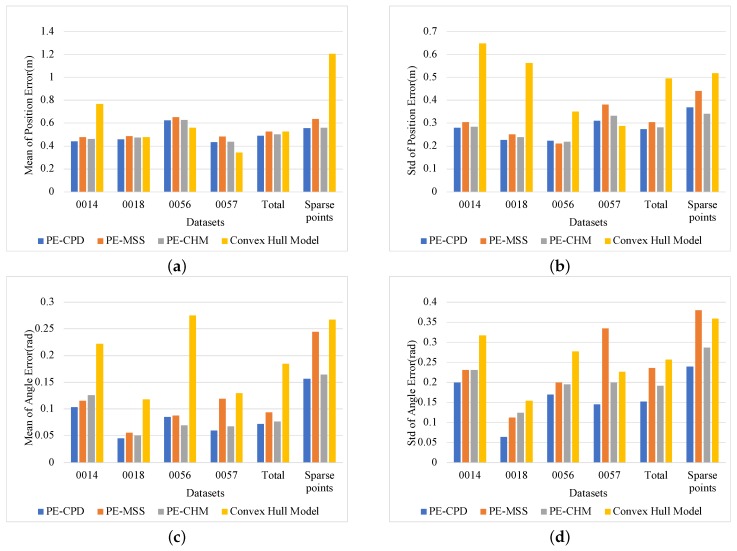
Statistical results of pose-estimation deviations. (**a**,**b**) Mean and standard deviation of position errors, respectively. (**c**,**d**) Mean and standard deviation of yaw angle errors, respectively.

In KITTI, four datasets, 0014, 0018, 0056, and 0057, were utilized to verify pose-estimation accuracy. These four datasets were collected from the city scenarios and contained many vehicles. The optical images of these datasets are shown in Figure 8. In addition to separately counting the estimation accuracy for each dataset, the average results of the deviations of the four datasets and the pose estimation of targets with sparse points, which means that the objects returned less than 50 points, were calculated at the same time. PE-MSS [22], PE-CPD [27], and the model-fitting method in [14] were implemented as the comparative experiments. The estimated poses of vehicles in the range of 80 m are utilized to perform the statistics. The provided labels in KITTI were employed to obtain the statistical deviations in position and angle, respectively, which are shown in Figure 9. The position deviation represents the distance between the midpoint of the ground truth and the midpoint of the fitting model. The angle deviation is the difference between the angle of ground truth and the angle of the estimated pose.

Figure 9a,b show statistical errors in position estimation. Among the four pose-estimation methods, the convex-hull model-fitting method in [14] had a large fluctuation on estimation accuracy, that is, it had well-estimated results in some scenarios (such as the 0057 dataset), but it performed poorly in other scenarios, such as the 0014 dataset and estimation with sparse point-clouds. The reason for this result is that the criteria for model fitting is single and cannot be adapted to the model fitting requirements for various scenarios. The center of the model is determined by the center of bounding box. For targets with sparse point-clouds and a missing contour, deviation between the position of the bounding box and the real target’s center is large. The proposed PE-CHM solves this problem by inferring the intersection with the two visible edges and using the fixed model size to determine the target’s position. Meanwhile, the proposed model-fitting criteria that compose four factors make the model adapt to the environment. The pose estimation of the three methods with fixed model size, namely, PE-CPD, PE-MSS, and PE-CHM, are basically stable for each dataset, showing robustness to the environment. The PE-CHM and PE-CPD algorithms had similar performance, and both had better estimation accuracy than PE-MSS, which indicates that, by introducing motion information, the target’s pose-estimation accuracy is improved, especially for a target with incomplete contour.

Figure 9c,d show the deviation results of the yaw angles. The disadvantages of the convex-hull model-fitting method using a single criterion for pose estimation are more obvious. Deviations between estimation and ground truth are large and estimations are unstable. The PE-MSS method performed badly in some situations, such as in the 0057 dataset and in the case with sparse point-clouds. The reason is that estimation with PE-MSS easily falls into local optimal estimation when vehicles are distant with sparse point-clouds and incomplete contours. The performances of the PE-CPD method and the proposed PE-CHM method were similar. The mean of the angular deviation of PE-CHM was 0.0079 rad larger than PE-CPD, and 0.0801 and 0.1033 rad smaller than PE-MSS and convex-hull model fitting, respectively.

**Figure 10:**
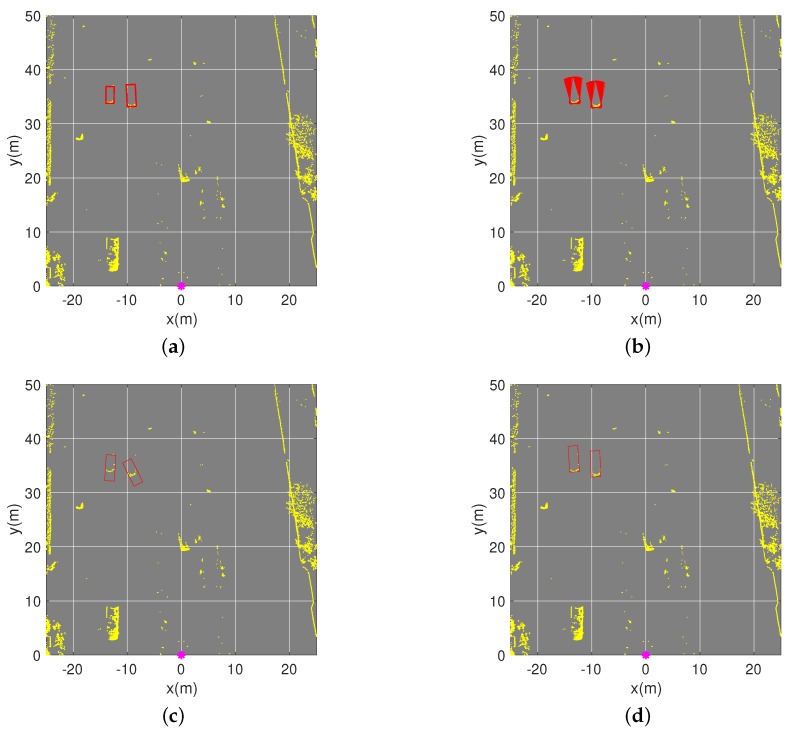
Pose estimation of two vehicles in the scenario. (**a**) Ground truth of their poses. (**b**–**d**) Estimated poses with the proposed pose estimation based on coherent point drift (PE-CPD), convex-hull model, and the proposed PE-CHM algorithm, respectively. Experiments were implemented one hundred times.

Figure 10 shows an example of the pose-estimation results of two vehicles with only one visible short edge. Since PE-CPD used random sampling to determine the best fitted model, their results of 100 pose estimations varied over a small interval. PE-CHM and the convex-hull model use the objective function to determine the best bounding box without random sampling, therefore they only obtain one estimated pose. It is shown in Figure 10c that using the center of the bounding box as the pose of the objects brings a big deviation between estimated position and actual position. With the multifactor objective function in the convex-hull model and the position-inference process, the proposed PE-CHM had accurate estimation.

### 4.2. Dynamic Vehicle Detection

In this section, two datasets in KITTI, 0018 and 0056, were applied to verify vehicle detection performance. The distance of 40 m was utilized to divide the detection range into distant area and close area. There are totally 816 targets in 0018 dataset, among which 448 targets are in the close area. The 0056 dataset contains 407 targets in close area and 22 targets in distant area. PE-MSS-based detection [22], PE-CPD-based detection [27], and the detection method that introduces the convex-hull model [14] into our detection system were utilized as the comparative experiments. Detection range was set to [0,80] (m) from the Lidar. The detailed detection results are shown in Table 1. $N_{D}$ and $N_{F}$ denotes the numbers of detected vehicles and false alarms, respectively. $N_{T}$ denotes the number of total vehicles in the scenarios. The precision *P* and recall *R* are calculated by Equation (15). As shown in Equation (16), the $F_{1}$ score is the harmonic average of *P* and *R*, which comprehensively represents the detection performance of the detection algorithm. The larger the value of $F_{1}$ score is, the better the detection performance will be.

(15) $$P=\frac{N_{D}}{N_{D}+N_{F}},R=\frac{N_{D}}{N_{T}}$$

(16) $$F_{1}=\frac{2PR}{P+R}.$$

Table 1 shows the detection results with the original convex-hull model and the PE-MSS-based method. The convex-hull method detected many false alarms from the scenarios, and the PE-MSS- based method had poor detection performance in the distant area. The convex-hull model that was proposed in [14] only used the sum of minimum distances from the bounding box as the objective function to determine the optimal pose, in which the center of the optimal bounding box was utilized as the object position. Since objects in the scenario could be occluded by obstacles, and the contour of the objects in the distant area was partially lost, the obtained center position’s change could cause a large deviation and the pose could be determined at a position with a large deviation on the yaw angle, which violates motion consistency and result in the loss of the vehicle. In the distant area, for objects with sparse point-clouds, the PE-MSS easily fell into the local extremum during pose estimation, resulting in wrong pose results. Inaccurate pose estimation also results in efficiency reduction in subsequent vehicle detection. For the close area, PE-MSS could have better pose estimation than the convex-hull model, so detection results were better.

Table 2 shows the results of the PE-CPD based method and the proposed PE-CHM-based method. Compared with the results in Table 1, both these methods had better detection performance in both the close and the distant area. Compared with the PE-CPD method, pose-estimation accuracy with the proposed PE-CHM was slightly reduced in the distant area, and the number of false alarms was slightly increased in the whole area. By comprehensively considering detection and false-alarm suppression performances, the $F_{1}$ scores of the PE-CPD method and the proposed method were 0.86 and 0.85, respectively, which indicates that the proposed method achieved similar detection performance to the PE-CPD method, offering the advantage of low computational complexity, which is explained in the next experiment.

### 4.3. Operation Time

In this work, all experiments were implemented on a MacBook Pro with a 2.9 GHz Intel Core i5 CPU and 8 GB main memory. Detection methods were conducted in MATLAB 2017b. Detection operation time with PE-CPD [10], and proposed detection with PE-CHM are listed in Table 3.

There are a total of 294 point-cloud frames in the 0056 dataset. The point-clouds were labeled with 0–293. The PE-CPD-based detection method utilized 150.9 s to finish detection, while PE-CHM-based detection reduced computation time by more than half. In frames 221–230, there was only a single vehicle in the scenario, and detailed statistics were recorded on the time consumption of each detection stage. It can be seen that the same preprocessing process had a similar operation time while ignoring the impact of the software. After removing the time consumption of the preprocessing process, PE-CPD needed an average of 98 ms to detect a point-cloud frame, and PE-CHM only needed 21 ms, which indicates that PE-CHM obviously improved pose-estimation efficiency.

## 5. Conclusions

To quickly and accurately detect dynamic vehicles in road scenarios, a novel pose-estimation method based on the convex-hull model and position inference was proposed in this paper. The main contributions are listed as follows.

First, multiple factors including the minimum boundary distance, minimum enclosed area, and minimum difference with the yaw angle, were utilized to construct the objective function for bounding box determination, which increases the adaptability of the convex-hull model to the environment. The construction of the convex-hull model is the foundation of the proposed pose estimation.

Second, a pose-estimation algorithm based on the convex-hull model and pose inference was proposed to estimate the pose of objects with a simple process. The proposed PE-CHM algorithm only uses contour point-clouds in the convex-hull set to determine the target pose, which reduces the amount of computation compared with the existing PE-CPD method.

Third, the proposed PE-CHM algorithm is applied to detect the dynamic vehicles in road scenarios. The experimental results showed that the proposed method could significantly reduce computation time while maintaining good detection efficiency.

## Figures and Tables

**Table 1:** Dynamic vehicle detection results by the convex-hull model method and pose estimation with modified scaling series (PE-MSS) method.

Datasets	Range	$N_{T}$	Convex Hull Method					PE-MSS Method				
			$N_{D}$	$N_{F}$	P	R	$F_{1}$	$N_{D}$	$N_{F}$	P	R	$F_{1}$
0018	<40 m	448	348	17	0.95	0.78	0.86	357	22	0.94	0.80	0.86
	>40 m	368	196	70	0.74	0.53	0.62	39	9	0.81	0.11	0.18
	Total	816	544	87	0.86	0.67	0.75	396	31	0.93	0.49	0.64
0056	<40 m	407	314	97	0.76	0.78	0.77	359	32	0.92	0.88	0.90
	>40 m	22	9	52	0.15	0.41	0.22	0	9	0	0	-
	Total	429	323	149	0.68	0.76	0.72	359	41	0.90	0.84	0.87
Overall	<40 m	855	662	114	0.85	0.67	0.75	716	54	0.93	0.84	0.88
	>40 m	390	205	248	0.45	0.53	0.49	39	18	0.68	0.10	0.17
	Total	1245	867	362	0.71	0.70	0.70	755	72	0.91	0.61	0.73

**Table 2:** Dynamic vehicle detection results by the pose estimation based on coherent point drift (PE-CPD) method and the proposed pose estimation with convex-hull model (PE-CHM) method.

Datasets	Range	$N_{T}$	PE-CPD Method					PE-CHM Method				
			$N_{D}$	$N_{F}$	P	R	$F_{1}$	$N_{D}$	$N_{F}$	P	R	$F_{1}$
0018	<40 m	448	368	5	0.99	0.82	0.90	374	10	0.97	0.83	0.89
	>40 m	368	219	16	0.93	0.60	0.73	207	16	0.93	0.56	0.70
	Total	816	587	21	0.97	0.72	0.82	581	26	0.96	0.71	0.82
0056	<40 m	407	385	21	0.95	0.95	0.95	377	31	0.92	0.94	0.93
	>40 m	22	9	3	0.75	0.41	0.53	8	8	0.50	0.36	0.42
	Total	429	394	24	0.94	0.92	0.93	385	39	0.91	0.91	0.91
Overall	<40 m	855	753	26	0.97	0.88	0.92	751	41	0.95	0.88	0.91
	>40 m	390	228	19	0.92	0.58	0.72	215	24	0.90	0.55	0.68
	Total	1245	981	45	0.96	0.79	0.86	966	65	0.94	0.78	0.85

**Table 3:** Operation-time comparison.

Methods	Total Time	Time/Frame	Frame 221–230					
			Time	Read Data	Ground Tracking	RBNN	Virtual Scan	Detection Time/Frame
PE-CPD based	150.9 s	513 ms	1976 ms	42 ms	60 ms	681 ms	203 ms	98 ms
PE-CHM based	69.6 s	237 ms	1125 ms	38 ms	49 ms	628 ms	196 ms	21 ms

## References

[1] Zou Y., Zhang W., Weng W., Meng Z. (2019). Multi-Vehicle Tracking via Real-Time Detection Probes and a Markov Decision Process Policy. Sensors.

[2] Velazquez-Pupo R., Sierra-Romero A., Torres-Roman D., Shkvarko Y., Santiago-Paz J., Gómez-Gutiérrez D., Robles-Valdez D., Hermosillo-Reynoso F., Romero-Delgado M. (2018). Vehicle detection with occlusion handling, tracking, and OC-SVM classification: A high performance vision-based system. Sensors.

[3] Guo Z., Cai B., Jiang W., Wang J. (2019). Feature-based detection and classification of moving objects using LiDAR sensor. IET Intell. Transp. Syst..

[4] Nguyen V.D., Nguyen T.T., Nguyen D.D., Lee S.J., Jeon J.W. (2013). A Fast Evolutionary Algorithm for Real-Time Vehicle Detection. IEEE Trans. Veh. Technol..

[5] Satzoda R.K., Trivedi M.M. (2016). Multipart Vehicle Detection Using Symmetry-Derived Analysis and Active Learning. IEEE Trans. Intell. Transp. Syst..

[6] Tian B., Li Y., Li B., Wen D. (2014). Rear-View Vehicle Detection and Tracking by Combining Multiple Parts for Complex Urban Surveillance. IEEE Trans. Intell. Transp. Syst..

[7] Stauffer C., Grimson W.E.L. (2000). Learning patterns of activity using real-time tracking. IEEE Trans. Pattern Anal. Mach. Intell..

[8] Yang B., Zhang S., Tian Y., Li B. (2019). Front-Vehicle Detection in Video Images Based on Temporal and Spatial Characteristics. Sensors.

[9] Ma Y., Anderson J., Crouch S., Shan J. (2019). Moving Object Detection and Tracking with Doppler LiDAR. Remote Sens..

[10] Liu K., Wang W., Tharmarasa R., Wang J. (2019). Dynamic Vehicle Detection With Sparse Point Clouds Based on PE-CPD. IEEE Trans. Intell. Transp. Syst..

[11] Sualeh M., Kim G.W. (2019). Dynamic Multi-LiDAR Based Multiple Object Detection and Tracking. Sensors.

[12] Yao W., Stilla U. (2011). Comparison of Two Methods for Vehicle Extraction From Airborne LiDAR Data Toward Motion Analysis. IEEE Geosci. Remote Sens. Lett..

[13] Börcs A., Nagy B., Benedek C. (2017). Instant Object Detection in Lidar Point Clouds. IEEE Geosci. Remote Sens. Lett..

[14] Börcs A., Nagy B., Baticz M., Benedek C. A model-based approach for fast vehicle detection in continuously streamed urban LIDAR point clouds. Proceedings of the Asian Conference on Computer Vision.

[15] Chavez-Garcia R.O., Aycard O. (2016). Multiple sensor fusion and classification for moving object detection and tracking. IEEE Trans. Intell. Transp. Syst..

[16] Zeng Y., Hu Y., Liu S., Ye J., Han Y., Li X., Sun N. (2018). RT3D: Real-Time 3-D Vehicle Detection in LiDAR Point Cloud for Autonomous Driving. IEEE Robot. Autom. Lett..

[17] Zhao J., Xu H., Liu H., Wu J., Zheng Y., Wu D. (2019). Detection and tracking of pedestrians and vehicles using roadside LiDAR sensors. Transp. Res. Part C Emerg. Technol..

[18] Börcs A., Benedek C. (2015). Extraction of Vehicle Groups in Airborne Lidar Point Clouds With Two-Level Point Processes. IEEE Trans. Geosci. Remote Sens..

[19] Fortin B., Lherbier R., Noyer J.C. (2012). Feature Extraction in Scanning Laser Range Data Using Invariant Parameters: Application to Vehicle Detection. IEEE Trans. Veh. Technol..

[20] Fortin B., Lherbier R., Noyer J.C. (2015). A Model-Based Joint Detection and Tracking Approach for Multi-Vehicle Tracking With Lidar Sensor. IEEE Trans. Intell. Transp. Syst..

[21] Kim D., Jo K., Lee M., Sunwoo M. (2017). L-shape model switching-based precise motion tracking of moving vehicles using laser scanners. IEEE Trans. Intell. Transp. Syst..

[22] Chen T., Wang R., Dai B., Liu D., Song J. (2016). Likelihood-Field-Model-Based Dynamic Vehicle Detection and Tracking for Self-Driving. IEEE Trans. Intell. Transp. Syst..

[23] Graham R.L. (1972). An efficient algorithm for determining the convex hull of a finite planar set. Inf. Process. Lett..

[24] Liu K., Wang W., Wang J. (2019). Pedestrian Detection with Lidar Point Clouds Based on Single Template Matching. Electronics.

[25] Arastounia M., Lichti D. (2015). Automatic object extraction from electrical substation point clouds. Remote Sens..

[26] Vaaja M., Kurkela M., Virtanen J.P., Maksimainen M., Hyyppä H., Hyyppä J., Tetri E. (2015). Luminance-corrected 3D point clouds for road and street environments. Remote Sens..

[27] Liu K., Wang W., Tharmarasa R., Wang J., Zuo Y. (2019). Ground Surface Filtering of 3D Point Clouds Based on Hybrid Regression Technique. IEEE Access.

[28] Zeng S. (2013). An object-tracking algorithm for 3-D range data using motion and surface estimation. IEEE Trans. Intell. Transp. Syst..

[29] Klasing K., Wollherr D., Buss M. A clustering method for efficient segmentation of 3D laser data. Proceedings of the IEEE International Conference on Robotics and Automation.

[30] Geiger A., Lenz P., Stiller C., Urtasun R. (2013). Vision meets robotics: The KITTI dataset. Int. J. Robot. Res..

